# Postoperative Impact of Anti‐Inflammatory Drug on Cancer Recurrence in Patients With Locally Advanced Gastric Cancer After Curative Gastrectomy: A Pilot Retrospective Study

**DOI:** 10.1002/ags3.70110

**Published:** 2025-10-12

**Authors:** Ryota Matsui, Manabu Ohashi, Motonari Ri, Rie Makuuchi, Masaru Hayami, Tomoyuki Irino, Takeshi Sano, Souya Nunobe

**Affiliations:** ^1^ Department of Gastroenterological Surgery The Cancer Institute Hospital of Japanese Foundation for Cancer Research Tokyo Japan; ^2^ Department of Gastrointestinal Surgery/Breast Surgery, Graduate School of Medical Science Kanazawa University Kanazawa Japan

**Keywords:** C‐reactive protein, gastric cancer, postoperative inflammation, prognosis, recurrence‐free survival

## Abstract

**Background:**

Elevated postoperative inflammation is associated with reduced recurrence‐free survival (RFS) after gastrectomy in patients with gastric cancer, independent of postoperative complications. The effect of the administration of anti‐inflammatory drugs immediately after gastrectomy on prolonging RFS in patients with gastric cancer has not been fully investigated. In this study, we aimed to investigate the effects of anti‐inflammatory drugs on the long‐term recurrence rates of gastric cancer after radical resection.

**Methods:**

This retrospective cohort study included consecutive patients who underwent radical gastrectomy for primary pStage II–III gastric cancer between May 2006 and March 2017. We performed propensity score matching using a logistic regression model to adjust for patient background, compared RFS, and used Cox proportional hazard regression to identify prognostic factors.

**Results:**

Median duration of follow‐up was 62 months. After matching, 591 patients were included in the untreated group, and 197 were included in the group treated with anti‐inflammatory drugs. RFS was no significant difference in the two groups (5‐year RFS: treated group, 73.2%; untreated group, 68.4%; *p* = 0.246), but peritoneal recurrence was significantly lower in the treated group (*p* = 0.028). Multivariate analyses showed that anti‐inflammatory drugs were independent prognostic factors for recurrence‐free survival (hazard ratio, 0.751; 95% confidence interval, 0.569–0.992; *p* = 0.044). In the subgroup analysis, using multivariate analysis for recurrence‐free survival, anti‐inflammatory drugs were more effective in patients with pStage III than in those with pStage II disease.

**Conclusion:**

Anti‐inflammatory drug administration after radical resection may prolong recurrence‐free survival in patients with pStage III gastric cancer.

## Graphical abstract

Anti‐inflammatory drugs may be effective in improving recurrence‐free survival after radical gastrectomy in patients with pStage II or III gastric cancer.

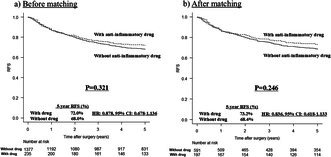

## 1 Introduction

Postoperative inflammation is associated with reduced recurrence‐free survival (RFS) after gastrectomy in patients with gastric cancer, independent of postoperative complications [1], although postoperative complications result in poor RFS after gastrectomy [2, 3]. Patients with advanced pathologically staged (pStage) gastric cancer may present with microscopic residual disease (MRD) in the blood, lymphatic system, abdominal cavity, liver, lungs, and bones. Postoperative inflammation promotes cancer differentiation in response to MRD and causes cancer adhesion to tissues [4, 5, 6]. Therefore, it is necessary to reduce postoperative inflammation at the tissue level to prolong RFS regardless of the presence or absence of postoperative complications.

Several reports on tissue inflammation have suggested that perioperative use of anti‐inflammatory drugs reduces the incidence of colorectal and lung cancer recurrence [7, 8, 9]. However, the impact of anti‐inflammatory drugs administered immediately after gastrectomy on RFS in patients with gastric cancer remains inadequately investigated. This hypothesis is based on the premise that suppression of postoperative inflammation by anti‐inflammatory agents may reduce cancer recurrence. In gastric cancer, adjuvant chemotherapy has also been shown to be effective in preventing cancer recurrence after radical resection [10, 11, 12]. Although postoperative adjuvant chemotherapy is recommended within 6 weeks after surgery, delayed introduction due to postoperative complications or poor dietary intake has been reported to increase cancer recurrence [13, 14, 15]. Therefore, in patients with advanced gastric cancer who are at a high risk of cancer recurrence after curative resection, it is postulated that anti‐inflammatory drugs should be administered immediately after surgery to reduce the risk of cancer recurrence independently of adjuvant chemotherapy.

This study aimed to investigate whether anti‐inflammatory drugs can prevent the recurrence of gastric cancer after radical resection. We hypothesized that anti‐inflammatory drugs would prolong RFS regardless of postoperative complications and that the effect would be greater in patients with more advanced pStage.

## 2 Methods

### 2.1 Eligibility Criteria

This retrospective cohort study was conducted at the Cancer Institute Hospital of the Japanese Foundation for Cancer Research and focused on patients with primary pStages II and III gastric cancer who underwent radical gastrectomy between May 2006 and March 2017. Patients diagnosed with gastric adenocarcinoma according to the Japanese Gastric Cancer Classification, 15th edition were included in this study [16]. TNM classification AND staging were defined according to the Japanese Gastric Cancer Classification, 15th edition. Patients with multiple cancers, non‐curative resections, remnant gastric cancer, those who regularly used anti‐inflammatory drugs or steroids, combined resection, and those receiving neoadjuvant chemotherapy or paraaortic lymph node dissection were excluded. Blood sample analyses, pathological results, and clinical data were retrospectively collected from the hospital's electronic medical records.

The Institutional Ethical Review Committee approved all experimental protocols used in this study (authorization number: 2024‐GB‐030) and the study was conducted in accordance with the Declaration of Helsinki and the Japan Ministry of Health, Labor, and Welfare's ethical guidelines for medical and health research involving human subjects. An opt‐out recruitment strategy was used to obtain consent, giving each patient the option to refuse participation.

### 2.2 Surgical Treatment and Adjuvant Chemotherapy

Patients with cT3 or deeper gastric cancer were recommended to undergo open gastrectomy, whereas patients with cT1 or cT2 gastric cancer were recommended to undergo laparoscopic gastrectomy. Various reconstruction techniques were used after the gastrectomies. Typically, Billroth I or Roux‐en‐Y was selected for distal gastrectomy, Roux‐en‐Y for total gastrectomy, gastrogastrostomy for pylorus‐preserving gastrectomy, and jejunal interposition or esophagogastrostomy for proximal gastrectomy.

After surgery, adjuvant chemotherapy with tegafur/gimeracil/oteracil (S‐1) or capecitabine with oxaliplatin (CAPOX) was initiated in patients with pStage II or III gastric cancer; doses were reduced in accordance with the guidelines if side effects were detected. S‐1 and CAPOX were administered for a maximum of 1 year and 6 months, respectively. No additional therapies were administered until recurrence occurred. The Japanese Gastric Cancer Treatment Guidelines were followed in the treatment of patients with relapses [13, 14].

### 2.3 Perioperative Management

The same clinical pathway was used for the perioperative care of each patient. Preoperative carbohydrate loading was part of the perioperative treatment. Every patient underwent mechanical bowel preparation the day before surgery. Prehabilitation was not provided. Rehabilitation was initiated on postoperative day (POD) 1. Patients not using antithrombotic drugs received epidural anesthesia for postoperative pain, while those using antithrombotic medication did not. On POD3, epidural anesthesia was eliminated. On POD 1, oral intake was initiated with water intake. On POD 3, the patient began eating solid food, first with rice gruel. On POD 4, they moved on to soft food, and on POD 5, they advanced to regular meal consumption in three steps.

### 2.4 Definitions of Anti‐Inflammatory Drug and Postoperative Inflammation

Patients who received nonsteroidal anti‐inflammatory drugs (NSAIDs) or acetaminophen for postoperative pain management, with or without epidural anesthesia, three to four times daily on a regular schedule for two or more consecutive days before discharge were classified as the anti‐inflammatory drug group (AID group). Patients who received NSAIDs or acetaminophen less than three times per day, for fewer than two consecutive days, or not at all were classified as the non‐anti‐inflammatory drug group (NAID group). After discharge, AID was prescribed for temporary use rather than as part of a regular regimen. The administration of all these drugs was based on the patients' symptoms and was not pre‐planned.

For the maximum value of postoperative CRP (CRPmax), we defined higher than 12.0 mg/dL as high, and lower than the cutoff value as low, according to the methods of a previous study [1].

### 2.5 Study Outcomes

The primary outcome was recurrence‐free survival (RFS), which was defined as the time between surgery and recurrence or death, whichever occurred first. We compared RFS before and after background adjustment using propensity scores.

### 2.6 Statistical Analysis

All statistical analyses were performed using EZR software (Saitama Medical Center, Jichi Medical University, Saitama, Japan). We performed propensity score matching using a logistic regression model to adjust for patient background and compare postoperative outcomes. The following covariates were matched: age, clinical stage (cStage), comorbidities, treatment period, surgical procedure, surgical approach, and lymph node dissection. For the matching process, the nearest‐neighbor method was used with a caliper size of 0.20. Patients were divided into two groups: NAID and AID, and the two groups were matched three‐to‐one. We compared the RFS in both groups before and after matching using the log‐rank test. Cox proportional hazards regression was used to identify prognostic factors and calculate hazard ratios (HRs). Comparisons between the two groups were conducted using the Mann–Whitney *U* test for continuous variables and the chi‐squared test or Fisher's exact test for categorical variables. Statistical significance was set at *p* < 0.05.

### 2.7 Other Parameters

Postoperative complications were assigned a Clavien–Dindo classification grade ≥ II if they occurred within 30 days after surgery [17]. We designated grade ≥ III of the Clavien–Dindo classification as a severe complication [17]. Infectious complications were defined as surgical site infection, intra‐abdominal abscess, and pneumonia.

Chronic kidney disease was defined as an estimated glomerular filtration rate of < 60 mL/min/1.73 m^2^. Diabetes was characterized by preoperative glycosylated hemoglobin levels ≥ 6.5% or a history of diabetes treatments. Chronic obstructive pulmonary disease was defined as forced expiratory volume1.0% < 70% on spirometry.

## 3 Results

### 3.1 Patient Characteristics

**FIGURE 1:**
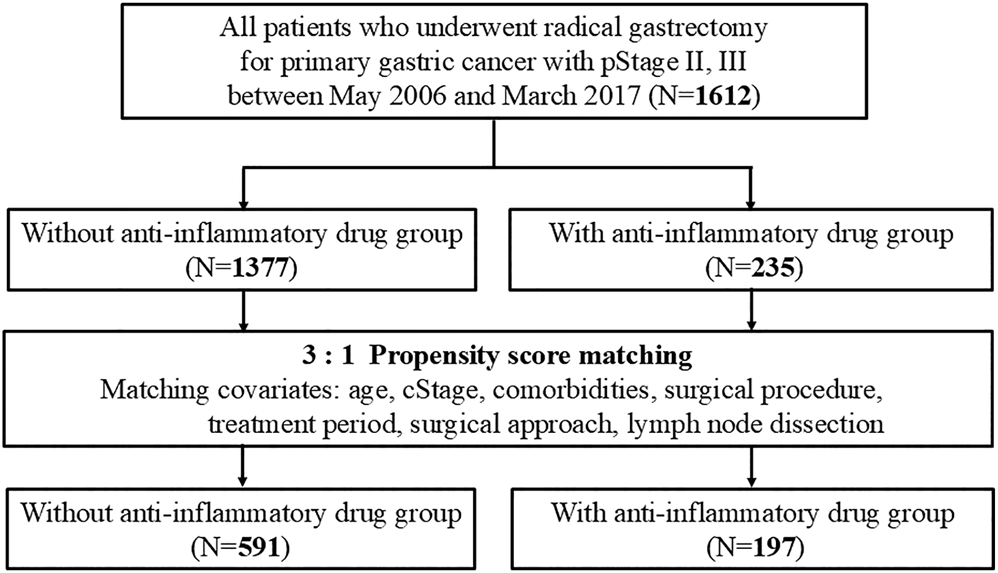
Study flowchart. We used propensity score matching to adjust for patient background.

A flowchart of the study is shown in Figure 1. Of the 1612 patients, 1377 (85.4%) and 235 (14.6%) were classified into the NAID and AID groups, respectively. The median duration of anti‐inflammatory drug use was 6 days (interquartile range: 5–7 days). After matching, 591 and 197 patients were assigned to NAID and AID groups, respectively. Patient backgrounds before and after matching are shown in Table 1. When compared, the AID group had significantly more severe complications than the NAID group (*p* = 0.012).

**TABLE 1:** Patient characteristics.

	Before matching			After matching		
	Without anti‐ inflammatory drug (*N* = 1377)	With anti‐ inflammatory drug (*N* = 235)	*p*	Without anti‐ inflammatory drug (*N* = 591)	With anti‐ inflammatory drug (*N* = 197)	*p*
Age (years), median (IQR)	65.0 (58.0, 73.0)	66.0 (55.0, 73.0)	0.458	65.0 (58.0, 73.0)	66.0 (55.0, 73.0)	0.458
Sex						
Male	876 (63.6%)	160 (68.1%)	0.211	371 (62.8%)	130 (66.0%)	0.442
Female	501 (36.4%)	75 (31.9%)		220 (37.2%)	67 (34.0%)	
BMI (kg/m^2^), median (IQR)	22.3 (20.1, 24.4)	22.9 (20.6, 24.8)	0.092	22.3 (20.1, 24.4)	22.8 (20.1, 24.5)	0.338
Nutrition related indicators						
PNI, median (IQR)	47.7 (44.1, 51.1)	47.8 (43.9, 51.2)	0.933	47.7 (44.1, 51.1)	47.8 (43.9, 51.2)	0.933
GNRI, median (IQR)	101.2 (94.7, 107.0)	102.3 (95.8, 108.2)	0.163	101.3 (95.3, 106.9)	102.2 (95.9, 107.6)	0.434
Comorbidity						
Chronic kidney disease	203 (14.8%)	32 (13.6%)	0.690	96 (16.3%)	25 (12.7%)	0.255
COPD	52 (3.8%)	12 (5.1%)	0.364	24 (4.1%)	10 (5.1%)	0.546
Diabetes	142 (10.3%)	36 (15.3%)	0.032	76 (12.9%)	28 (14.2%)	0.628
Antiplatelet drug use	10 (0.7%)	1 (0.4%)	1.000	2 (0.3%)	0 (0%)	1.000
Clinical stage						
I	402 (29.2%)	67 (28.5%)	< 0.001	173 (29.3%)	56 (28.4%)	0.988
II	338 (24.5%)	88 (37.4%)		197 (33.3%)	68 (34.5%)	
III	595 (43.2%)	77 (32.8%)		212 (35.9%)	70 (35.5%)	
IVA	42 (3.1%)	3 (1.3%)		9 (1.5%)	3 (1.5%)	
Surgical approach						
Laparoscopic surgery	185 (13.4%)	44 (18.7%)	0.043	103 (17.4%)	36 (18.3%)	0.829
Open surgery	1192 (86.6%)	191 (81.3%)		488 (82.6%)	161 (81.7%)	
Surgical procedure						
Distal gastrectomy	845 (61.4%)	135 (57.4%)	0.278	342 (57.9%)	117 (59.4%)	0.739
Total gastrectomy	532 (38.6%)	100 (42.6%)		249 (42.1%)	80 (40.6%)	
Lymph node dissection						
D1+	159 (11.5%)	21 (8.9%)	0.264	65 (11.0%)	21 (10.7%)	1.000
D2	1218 (88.5%)	214 (91.1%)		526 (89.0%)	176 (89.3%)	
Splenectomy	195 (14.2%)	25 (10.6%)	0.181	66 (11.2%)	20 (10.2%)	0.792
CRP (mg/dL), median (IQR)						
POD1	8.4 (6.0, 11.0)	8.3 (5.7, 10.8)	0.517	8.4 (6.0, 10.9)	8.3 (5.7, 10.8)	0.517
POD3	12.4 (8.7, 17.0)	13.5 (9.4, 18.4)	0.041	12.4 (8.7, 17.0)	13.5 (9.4, 18.4)	0.041
POD5	6.4 (3.9, 10.4)	8.7 (4.9, 14.1)	< 0.001	6.4 (3.9, 10.4)	8.7 (4.9, 14.1)	< 0.001
Pathological stage						
II	678 (49.2%)	105 (44.7%)	0.204	273 (46.2%)	95 (48.2%)	0.622
III	699 (50.8%)	130 (55.3%)		318 (53.8%)	102 (51.8%)	
Histological type						
Tub1, Tub2, Pap	489 (35.5%)	75 (31.9%)	0.301	220 (37.2%)	66 (33.5%)	0.392
Por, Sig	888 (64.5%)	160 (68.1%)		371 (62.8%)	131 (66.5%)	
Treatment period						
Until 2011	503 (36.5%)	0 (0%)	< 0.001	0 (0%)	0 (0%)	1.000
Since 2011	874 (63.5%)	235 (100%)		591 (100%)	197 (100%)	
Postoperative complications						
Overall complications	329 (23.9%)	82 (34.9%)	< 0.001	164 (27.7%)	66 (33.5%)	0.125
Infectious complications	248 (18.0%)	59 (25.1%)	0.015	113 (19.1%)	49 (24.9%)	0.085
Severe complications	109 (7.9%)	39 (16.6%)	< 0.001	55 (9.3%)	32 (16.2%)	0.012
Incisional SSI	62 (4.5%)	13 (5.5%)	0.502	28 (4.7%)	13 (6.6%)	0.354
Abdominal abscess	159 (11.5%)	34 (14.5%)	0.231	70 (11.8%)	27 (13.7%)	0.531
Anastomotic leakage	36 (2.6%)	12 (5.1%)	0.058	19 (3.2%)	9 (4.6%)	0.377
Pancreatic leakage	88 (6.4%)	13 (5.5%)	0.771	38 (6.4%)	9 (4.6%)	0.389
Pneumonia	40 (2.9%)	12 (5.1%)	0.106	22 (3.7%)	10 (5.1%)	0.407
Adjuvant chemotherapy	873 (63.4%)	153 (65.1%)		364 (61.6%)	122 (61.9%)	
S‐1	842 (61.1%)	116 (49.4%)	0.660	340 (57.5%)	114 (57.9%)	1.000
CAPOX	31 (2.3%)	37 (15.7%)	< 0.001	24 (4.1%)	8 (4.1%)	1.000

Abbreviations: BMI, body mass index; COPD, chronic obstructive pulmonary disease; CRP, C‐reactive protein; GNRI, geriatric nutritional risk index; IQR, interquartile range; Pap, papillary adenocarcinoma; PNI, prognostic nutritional index; POD, postoperative day; Por, poorly differentiated adenocarcinoma; Sig, signet‐ring cell carcinoma; SSI, surgical site infection; Tub1, well differentiated tubular adenocarcinoma; Tub2, moderately differentiated tubular adenocarcinoma.

### 3.2 Comparison of RFS Before and After Matching

**FIGURE 2:**
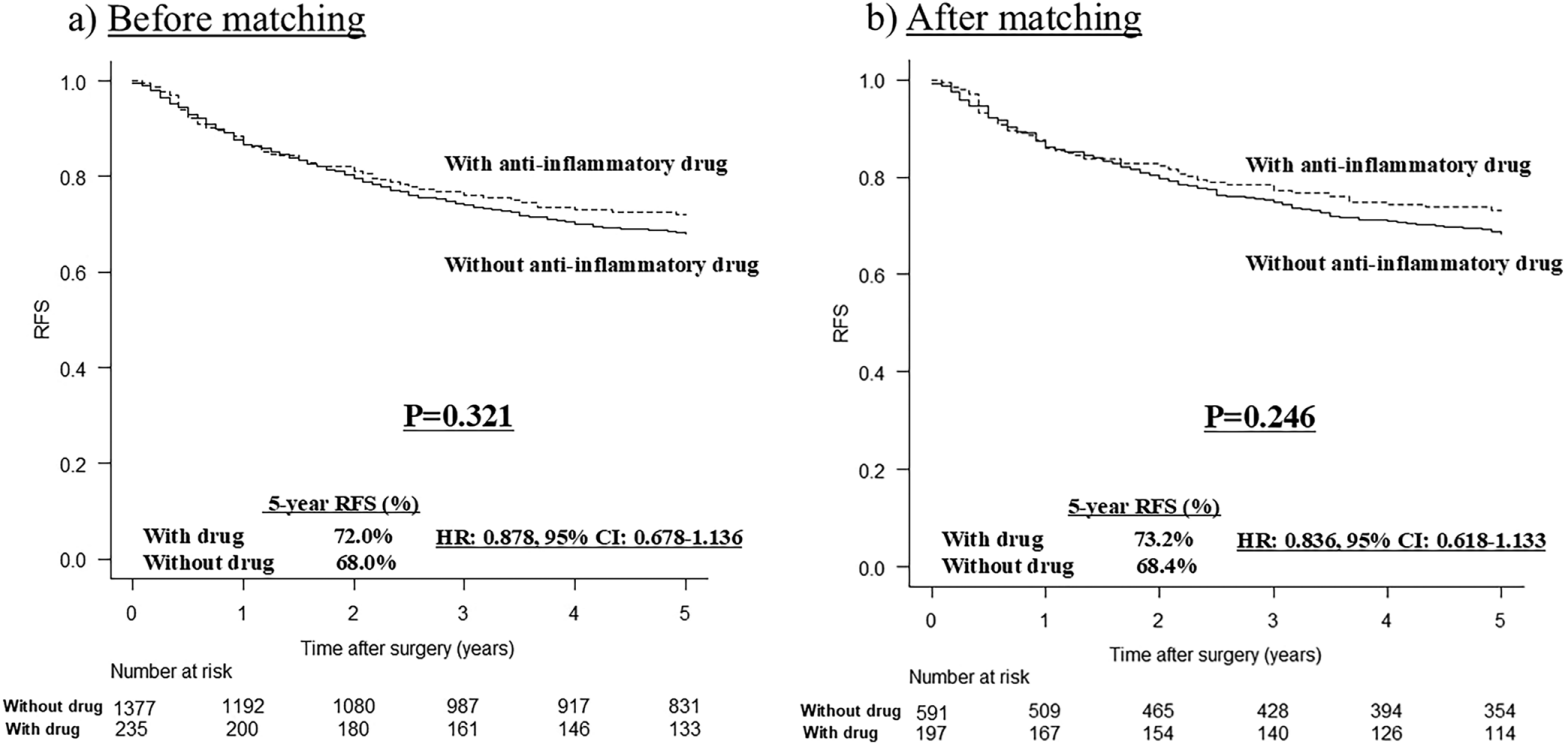
Comparison of RFS in patients with pStage II and III gastric cancer before and after matching according to the use of anti‐inflammatory drugs. (a) RFS did not differ significantly between the two groups before matching (*p* = 0.321). (b) The difference in RFS between the two groups increased after matching, but the difference was not significant (*p* = 0.246). RFS, recurrence‐free survival.

The median follow‐up period was 62 months (interquartile range = 40–77 months). A comparison of RFSs before and after matching is shown in Figure 2. RFS did not differ significantly between the two groups before matching (HR: 0.878, 95% confidence interval (CI): 0.678–1.136, *p* = 0.321; Figure 2a), although the RFS curve of the AID group was superior to that of the NAID group (5‐year RFS: 72.0% vs. 68.0%, respectively). The difference in survival curves between the NAID and AID groups increased after matching (5‐year RFS: 68.4% vs. 73.2%, respectively), but the difference was not significant (HR: 0.836, 95% CI: 0.618–1.133, *p* = 0.246; Figure 2b).

### 3.3 Comparison of Recurrence Patterns

Table 2 shows a comparison of recurrence patterns. After matching, peritoneal metastasis was significantly lesser in the AID group than in the NAID group (*p* = 0.028).

**TABLE 2:** Comparison of recurrence patterns.

	Before matching			After matching		
	Without anti‐inflammatory drug (*N* = 1377)	With anti‐inflammatory drug (*N* = 235)	*p*	Without anti‐inflammatory drug (*N* = 591)	With anti‐inflammatory drug (*N* = 197)	*p*
Recurrence	368 (26.7%)	50 (21.3%)	0.091	155 (26.2%)	38 (19.3%)	0.056
Hematogenous metastasis	130 (9.4%)	17 (7.2%)	0.327	46 (7.8%)	13 (6.6%)	0.642
Peritoneal metastasis	156 (11.3%)	22 (9.4%)	0.431	77 (13.0%)	14 (7.1%)	0.028
Lymph node metastasis	81 (5.9%)	16 (6.8%)	0.554	32 (5.4%)	14 (7.1%)	0.383
Local recurrence	21 (1.5%)	1 (0.4%)	0.235	10 (1.7%)	1 (0.5%)	0.308
Recurrence within 1 year	159 (11.5%)	27 (11.5%)	1.000	69 (11.7%)	23 (11.7%)	1.000

### 3.4 Prognostic Factors for RFS by Multivariate Analysis

Table 3 shows the results of univariate and multivariate analyses for RFS in all patients. The use of anti‐inflammatory drugs was an independent prognostic factor for RFS (HR: 0.751, 95% CI: 0.569–0.992, *p* = 0.044). High CRPmax was also an independent poor prognostic factor (HR, 1.215; 95% CI, 1.011–1.461; *p* = 0.038).

**TABLE 3:** Results of analysis of prognostic factors for recurrence‐free survival in patients with pStage II, III.

Variables	Univariate analysis			Multivariate analysis		
	HR	95% CI	*p*	HR	95% CI	*p*
Age (years) ≥ 70 (vs. < 70)	1.791	1.523–2.106	< 0.001	1.438	1.189–1.739	< 0.001
Age (years) ≥ 80 (vs. < 80)	2.280	1.807–2.877	< 0.001	1.666	1.252–2.218	< 0.001
Sex male (vs. female)	1.277	1.075–1.516	0.005	1.306	1.081–1.577	0.006
BMI (kg/m^2^) < 18.5 (vs. ≥ 18.5)	1.297	1.027–1.637	0.029	1.194	0.931–1.531	0.163
BMI (kg/m^2^) ≥ 25.0 (vs. < 25.0)	0.907	0.740–1.111	0.345	0.946	0.766–1.169	0.609
Chronic kidney disease present (vs. absent)	1.248	1.006–1.548	0.044	1.009	0.802–1.269	0.938
COPD present (vs. absent)	1.382	0.952–2.008	0.089	1.103	0.752–1.618	0.617
Diabetes present (vs. absent)	0.866	0.648–1.157	0.330	0.824	0.613–1.107	0.198
Surgical procedure total gastrectomy (vs. distal gastrectomy)	1.668	1.421–1.958	< 0.001	1.465	1.238–1.733	< 0.001
Surgical approach open (vs. laparoscopy)	2.031	1.524–2.706	< 0.001	1.362	0.951–1.950	0.091
Lymph node dissection D2 (vs. D1+)	0.914	0.712–1.173	0.478	0.594	0.439–0.804	< 0.001
pStage III (vs. II)	2.625	2.210–3.119	< 0.001	2.490	2.042–3.037	< 0.001
Histological type Por/Sig (vs. Tub1/Tub2, Pap)	0.906	0.768–1.069	0.243	1.062	0.893–1.262	0.496
Period since 2011 (vs. until 2011)	0.773	0.654–0.914	0.003	0.841	0.704–1.005	0.056
CRPmax ≥ 12 mg/dL present (vs. absent)	1.335	1.131–1.576	< 0.001	1.215	1.011–1.461	0.038
Anti‐inflammatory drug present (vs. absent)	0.878	0.678–1.136	0.323	0.751	0.569–0.992	0.044
Severe complication present (vs. absent)	1.473	1.140–1.902	0.003	1.244	0.953–1.625	0.109
Adjuvant chemotherapy present (vs. absent)	0.925	0.788–1.086	0.341	0.781	0.646–0.945	0.011

Abbreviations: BMI, body mass index; CI, confidence interval; COPD, chronic obstructive pulmonary disease; CRP, C‐reactive protein; HR, hazard ratio; Pap, papillary adenocarcinoma; POD, postoperative day; Por, poorly differentiated adenocarcinoma; Sig, signet‐ring cell carcinoma; Tub1, well differentiated tubular adenocarcinoma; Tub2, moderately differentiated tubular adenocarcinoma.

**FIGURE 3:**
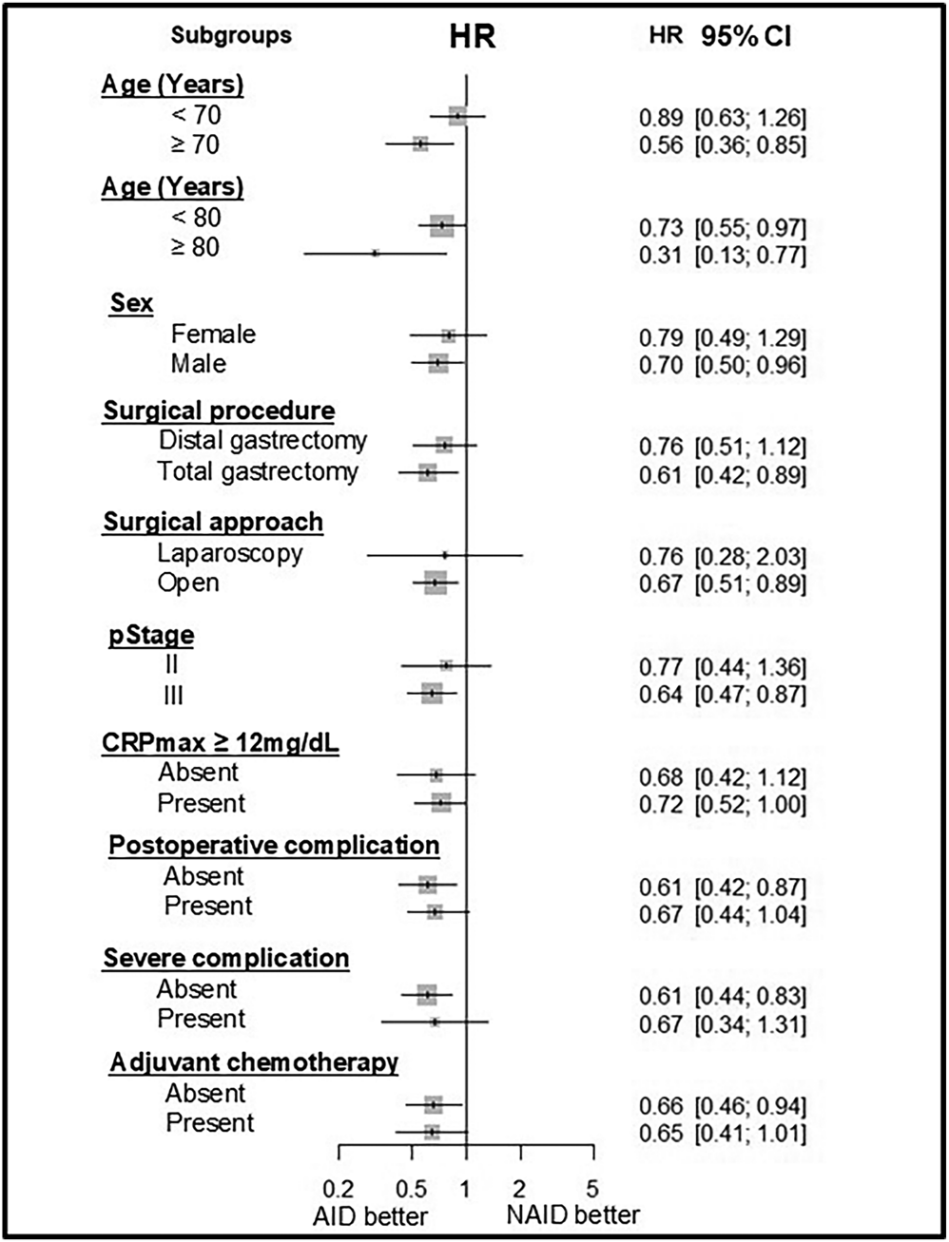
Subgroup differences in the effect of anti‐inflammatory drugs using multivariate analysis for RFS. Anti‐inflammatory drugs were more effective in patients with pStage III than pStage II, those undergoing open surgery rather than laparoscopic surgery, and total gastrectomy rather than distal gastrectomy, and in patients without postoperative complications, and ≥ 70 years.

Figure 3 shows the subgroup differences in the effects of anti‐inflammatory drugs on RFS using multivariate analysis. In patients ≥ 70 years of age, ≥ 80 years of age, who underwent total gastrectomy, had open surgery, had pStage III, and did not experience postoperative complications, the use of anti‐inflammatory drugs significantly favored survival.

## 4 Discussion

This study used adjusted comparisons by propensity score matching and multivariate analysis by Cox proportional hazards regression to evaluate the effects of anti‐inflammatory drugs on RFS after radical resection in patients with pStage II or III gastric cancer. In comparison of RFS after matching, the RFS of the AID group was superior to that of the NAID group, but the difference was not significant. In the recurrence pattern, peritoneal metastasis was significantly lesser in the AID group than in the NAID group after matching. In multivariate analysis, the use of anti‐inflammatory drugs was an independent favorable prognostic factor for RFS. Additionally, there were two informative findings in this study. First, subgroup analysis of the effect of anti‐inflammatory drugs on RFS using multivariate analysis showed the use of anti‐inflammatory drugs significantly favored survival in the group of patients ≥ 70 years of age, who underwent total gastrectomy, had open surgery, had pStage III, and did not develop postoperative complications. Second, multivariate analysis showed that a high CRPmax was an independent poor prognostic factor for RFS, regardless of the use of anti‐inflammatory drugs. These findings indicated that the use of anti‐inflammatory drugs immediately after surgery independently improved RFS, particularly in older patients with advanced‐stage cancer and in those who underwent gastrectomy and did not experience postoperative adverse events, which are associated with severe inflammation. Because a high CRPmax is associated with decreased RFS, reducing inflammation using anti‐inflammatory drugs may prolong survival.

This study used propensity score matching and multivariate analyses with the Cox proportional hazards regression model. Propensity score matching is advantageous because it allows simple comparisons after matching for patient backgrounds. However, a major disadvantage is that it cannot adjust for postoperative factors. After matching, we observed a significantly increased severe complications in the AID group. This may have had a negative effect on RFS; however, the survival curves tended to be favorable in the AID group. Because multivariate analysis can adjust for postoperative factors, anti‐inflammatory drugs were identified as independent favorable prognostic factors. This was the result of adjustment for postoperative factors.

High CRPmax was an independent poor prognostic factor for RFS in patients with pStage II or III gastric cancer. In patients with locally advanced cancer, MRD may persist after curative surgery, and postoperative inflammation can promote MRD growth and tissue adhesion [4, 5, 6]. Increased postoperative inflammation has promoted cancer recurrence and reduced RFS [1], which is consistent with the results of this study. Therefore, efforts to reduce postoperative inflammation, including the reduction of postoperative complications that cause inflammation, and the use of anti‐inflammatory drugs, should be considered to prolong long‐term survival.

Subgroup differences in the effect of anti‐inflammatory drugs on RFS using multivariate analysis showed that anti‐inflammatory drugs were more effective in older patients than in younger patients, in men than in women, in total gastrectomy than in distal gastrectomy, in open surgery than in laparoscopic surgery, in patients with pStage III than in those with pStage II, and in patients without postoperative complications. The proportion of patients receiving adjuvant chemotherapy in this study was significantly higher in patients aged < 70 years (72.7%) compared with those aged ≥ 70 years (46.8%). In addition, the proportion of adjuvant chemotherapy was significantly lower in patients aged ≥ 80 years (15.0%) than in those aged < 80 years (68.3%). Therefore, anti‐inflammatory drugs may be more effective in preventing cancer recurrence in older patients who are less likely to receive adjuvant chemotherapy. The CRP levels on POD1 or POD3 were significantly higher in open surgery than in laparoscopic surgery; similarly, CRP levels were significantly higher in total gastrectomy than in distal gastrectomy and were higher in men than in women. These findings suggest that anti‐inflammatory drugs may be more effective in patients with more severe postoperative inflammation. In the subgroup with postoperative complications, the use of anti‐inflammatory drugs reduced the HR but did not reach statistical significance. This finding may indicate that patients with postoperative complications had such severe inflammation that it was not reduced by anti‐inflammatory drugs. In contrast, anti‐inflammatory drugs effectively prolonged RFS in patients without postoperative complications, indicating that the use of anti‐inflammatory drugs may sufficiently reduce postoperative inflammation when complications do not occur. Previous reports have demonstrated that high postoperative inflammation is associated with reduced RFS even without postoperative complications [1]. Anti‐inflammatory drugs may be effective in preventing cancer recurrence in patients who are unable to receive adjuvant chemotherapy as an alternative treatment or who suffer from higher inflammation from their disease status and procedures. However, severe inflammation induced by surgical complications may not be overcome by anti‐inflammatory drugs.

The mechanisms by which postoperative inflammation promotes cancer progression and reduces RFS and by which anti‐inflammatory drugs counteract this are not fully understood. Several hypothesized mechanisms underly postoperative tumor progression. First, cytokine exposure from postoperative inflammation enables MRD growth. Cytotoxic mediators such as interleukin‐1 (IL‐1), IL‐6, and tumor necrosis factor‐alpha released in large amounts during the inflammatory response promote the growth and invasion of MRD, resulting in tumor recurrence and metastasis [18, 19, 20]. This mechanism, by which factors are released by immune‐competent cells during infection or surgical invasion, is the one affected by anti‐inflammatory drugs. Second, severe or persistent postoperative inflammation causes immunosuppression of the host. Persistent inflammation leads to an anti‐inflammatory immunosuppressed state, which is called immuno‐paralysis or anti‐inflammatory response syndrome (CARS) [18, 21]. In this state of immunosuppression, anti‐inflammatory cytokines such as IL‐10, which suppress antitumor immunity, are increased, cytokines such as IL‐12, which activate antitumor immunity, are decreased, and immunosuppressive cells such as regulatory T cells are increased, resulting in accelerated tumor growth [18]. Immune responses involving cytotoxic T lymphocytes and natural killer cells are also impaired by systemic inflammation and surgical stress, suppressing the response to MRD [3, 18]. Immunonutrition promotes postoperative recovery from intense inflammation, resulting in immune recovery against surgical invasion [22]. However, further basic science and clinical studies are needed to clarify the mechanisms of postoperative inflammation and tumor growth.

In the recurrence pattern after matching, peritoneal metastasis was significantly lower in the AID group, but there was no difference in hematogenous metastasis, lymph node metastasis, or local recurrence. The mechanism of peritoneal metastasis involves cancer cells spreading into the abdominal cavity and then adhering to tissues through adhesion molecules [23]. When inflammatory cytokines are induced by surgical invasion or inflammation, the expression of adhesion molecules in mesothelial cells increases, promoting peritoneal metastasis [23]. AID may inhibit tissue adhesion by suppressing the production and activity of these cytokines. Further basic research is needed to clarify this mechanism.

The proportion of adjuvant chemotherapy administration in this study was 63.6%. Among patients who did not receive adjuvant chemotherapy, the main reasons for not administering adjuvant chemotherapy were pT1 (25.9%), pT3N0 (23.7%), and age ≥ 80 years (21.3%), although all of these patients were pathologically Stage II or III gastric cancer. In other words, the reasons primarily involved patients for whom adjuvant chemotherapy is not recommended by clinical guidelines or elderly patients for whom there is insufficient supporting evidence. Thus, approximately 90% of patients received treatment in accordance with the guidelines, while a small proportion could not undergo adjuvant chemotherapy due to postoperative nutritional status or complications. This study found no significant difference in the efficacy of anti‐inflammatory drugs with or without adjuvant chemotherapy; however, this analysis included patients who were not eligible for adjuvant chemotherapy, as described above. On the other hand, anti‐inflammatory drugs appeared to be more effective in elderly patients aged ≥ 80 years, suggesting potential benefit in populations not eligible for adjuvant chemotherapy.

The present study has some limitations. First, this was a single‐center retrospective study. A prospective multicenter study should be conducted to demonstrate the universality of these results. Second, the mechanism by which postoperative inflammation promotes cancer progression and reduces RFS, and by which anti‐inflammatory drugs are effective against this mechanism, is not clear. Further basic science and clinical research is required to clarify these mechanisms. Third, in PSM, confounding factors such as performance status, comorbidities, and nutritional status may not have been fully controlled for. Fourth, patients who received neoadjuvant chemotherapy were excluded from this study. At our institution, indications for neoadjuvant chemotherapy include para‐aortic lymph node metastasis, bulky N2 disease, and liver metastases with three or fewer lesions. We excluded these patients because their treatment options differ significantly from the standard treatment, and their number was very small (less than 1%). Despite these limitations, this is the first study to show that the use of anti‐inflammatory drugs immediately after radical resection prolongs RFS in patients with advanced gastric cancer. The strengths of this study are the large sample size and the use of previously validated cutoff values for postoperative CRP. Studies are required to determine whether further suppression of postoperative inflammation with a combination of immunonutrition, laparoscopic surgery, and anti‐inflammatory drugs leads to improved long‐term survival.

## 5 Conclusion

The use of anti‐inflammatory drugs after radical resection may improve the RFS in patients with pStage II or III gastric cancer. This effect is more pronounced in older men who have undergone open or total gastrectomy, have pStage III gastric cancer, and have experience no postoperative complications. Although further evidence regarding the relationship between inflammation and oncological outcomes is required, the results of this study are useful for future anti‐inflammatory interventions in cancer treatments.

## Author Contributions

**Ryota Matsui:** conceptualization, investigation, writing – original draft, methodology, visualization, writing – review and editing, formal analysis, data curation. **Manabu Ohashi:** conceptualization, investigation, writing – original draft, methodology, validation, writing – review and editing, visualization, supervision, data curation, formal analysis. **Motonari Ri:** writing – original draft, methodology, validation, writing – review and editing. **Rie Makuuchi:** writing – original draft, methodology, validation, writing – review and editing. **Masaru Hayami:** writing – original draft, methodology, validation, writing – review and editing. **Tomoyuki Irino:** writing – original draft, methodology, validation, writing – review and editing. **Takeshi Sano:** writing – original draft, methodology, validation, writing – review and editing, supervision. **Souya Nunobe:** writing – original draft, methodology, validation, writing – review and editing, supervision.

## Ethics Statement

All procedures followed were in accordance with the ethical standards of the responsible committee on human experimentation (institutional and national) and with the Declaration of Helsinki of 1964 and its later versions. Informed consent to be included in the study, or the equivalent, was obtained from all patients. (authorization number: 2024‐GB‐030).

## Conflicts of Interest

The authors declare no conflicts of interest.

## Data Availability

The datasets generated and/or analyzed in the current study are available from the corresponding author upon reasonable request.
